# Novel AlkB Dioxygenases—Alternative Models for *In Silico* and *In Vivo* Studies

**DOI:** 10.1371/journal.pone.0030588

**Published:** 2012-01-24

**Authors:** Damian Mielecki, Dorota Ł. Zugaj, Anna Muszewska, Jan Piwowarski, Aleksandra Chojnacka, Marcin Mielecki, Jadwiga Nieminuszczy, Marcin Grynberg, Elżbieta Grzesiuk

**Affiliations:** Institute of Biochemistry and Biophysics, Polish Academy of Sciences, Warszawa, Poland; University of Massachusetts Medical School, United States of America

## Abstract

**Background:**

ALKBH proteins, the homologs of *Escherichia coli* AlkB dioxygenase, constitute a direct, single-protein repair system, protecting cellular DNA and RNA against the cytotoxic and mutagenic activity of alkylating agents, chemicals significantly contributing to tumor formation and used in cancer therapy. *In silico* analysis and *in vivo* studies have shown the existence of AlkB homologs in almost all organisms. Nine AlkB homologs (ALKBH1–8 and FTO) have been identified in humans. High ALKBH levels have been found to encourage tumor development, questioning the use of alkylating agents in chemotherapy. The aim of this work was to assign biological significance to multiple AlkB homologs by characterizing their activity in the repair of nucleic acids in prokaryotes and their subcellular localization in eukaryotes.

**Methodology and Findings:**

Bioinformatic analysis of protein sequence databases identified 1943 AlkB sequences with eight new AlkB subfamilies. Since *Cyanobacteria* and *Arabidopsis thaliana* contain multiple AlkB homologs, they were selected as model organisms for *in vivo* research. Using *E. coli alkB* ^−^ mutant and plasmids expressing cyanobacterial AlkBs, we studied the repair of methyl methanesulfonate (MMS) and chloroacetaldehyde (CAA) induced lesions in ssDNA, ssRNA, and genomic DNA. On the basis of GFP fusions, we investigated the subcellular localization of ALKBHs in *A. thaliana* and established its mostly nucleo-cytoplasmic distribution. Some of the ALKBH proteins were found to change their localization upon MMS treatment.

**Conclusions:**

Our *in vivo* studies showed highly specific activity of cyanobacterial AlkB proteins towards lesions and nucleic acid type. Subcellular localization and translocation of ALKBHs in *A. thaliana* indicates a possible role for these proteins in the repair of alkyl lesions. We hypothesize that the multiplicity of ALKBHs is due to their involvement in the metabolism of nucleo-protein complexes; we find their repair by ALKBH proteins to be economical and effective alternative to degradation and *de novo* synthesis.

## Introduction

Alkylating agents are a group of chemicals ubiquitous in the environment, which significantly contribute to tumor formation in humans but are also used in clinical settings. Chemicals, such as chlorambucil, cyclophosphamide, mitomycin C and cisplatin are exploited in cancer therapy [1]. Alkylating agents react with nucleic acid bases creating products that are either toxic, mutagenic or neutral to cells [2]. To minimize the consequences of the presence of alkylated bases in DNA, organisms evolved a variety of mechanisms for repair of these cytotoxic and/or mutagenic lesions. *Escherichia coli* AlkB protein (EcAlkB) is one of the four proteins (Ada, AlkA, AlkB, and AidB) induced within adaptive response to the presence of alkylating agents [3], [4]. It belongs to the dioxygenase family that uses non-heme Fe (II) and cofactors 2-oxoglutarate (2OG) and oxygen (O_2_) to initiate oxidative demethylation of DNA bases [5]. EcAlkB in the presence of O_2_ converts 2OG to succinate and CO_2_. The initial hydroxylation of the methyl group at the N^1^ position of adenine and N^3^ position of cytosine results in cleavage of the C-N bond restoring unmodified A and C bases in DNA [6], [7]. N^1^ of A and N^3^ of C are much more susceptible to methylation in single-stranded (ss) than in double-stranded (ds) DNA, and consequently AlkB repairs lesions 5–10-fold more efficiently in ssDNA. 1meA and 3meC lesions placed in RNA are also substrates for AlkB [8], [9]. The AlkB protein has been found to oxidize ethyl, propyl, hydroxyethyl, hydroxypropyl, and exocyclic ethano and etheno groups (1,*N* ^6^-ethenoadenine, 3,*N* ^4^ –ethenocytosine) added to DNA bases [10]–[12]. Alkylated DNA bases, 3-methylthymine (3meT) and 1-methylguanine (1meG) are also repaired by AlkB, but much less efficiently than 1meA or 3meC [13], [14].

Nine human EcAlkB homologs have been identified: ALKBH1–8 and FTO [15], [16], all containing a conserved 2-oxoglutarate -Fe(II) dioxygenase domain. Homologs 1, 2, and 3 possess demethylase activity typical for EcAlkB [8], [9], [17]. Recent data report dioxygenase activity also for homologs 4 and 5, but in these cases the ability to demethylate nucleic acids has not yet been shown [18], [19]. Two homologs, 1 and 8, show activities not found in any other AlkBs. ALKBH1 is a DNA lyase active at AP sites and does not require iron and 2-oxoglutarate, which indicates an active site distinct from the one for demethylation [20]. In contrast, ALKBH8 shows methyltransferase activity, modifying uridine in the wobble position of tRNA [21]. ALKBH8 consists of three domains, i.e. an N-terminal RNA recognition motif (RRM), followed by a characteristic AlkB-like dioxygenase domain, and a C-terminal methyltransferase (MT) domain. The MT domain of *Arabidopsis thaliana* At1g36310 denoted as AtTRM9 has been recently shown to represent the functional homolog of *Saccharomyces cerevisiae* Trm9 protein and be responsible for 5-methoxycarbonylmethyluridine (mcm^5^U) formation. Moreover, the protein AtALKBH8 displays high similarity to the N-terminal part of human ALKBH8, encompassing the RRM and AlkB domains, and is required for hydroxylation of mcm^5^U to (S) mcm^5^U in tRNA_UCC_ [22].

Mutations in the region of the gene encoding the FTO protein, the ninth AlkB homolog discovered, result in obesity [23]; however, the molecular basis of this phenomenon is unknown [24]. Biochemical activity of FTO was found to resemble EcAlkB since it is able to demethylate 1meA/3meC [16]. The very recent findings by Jia et al. [25] show that searching for new FTO substrates has just begun.

At least three homologs, ALKBH2, 3 and 8, are affected in various cancers/tumors. Upregulation of ALKBH2 and 3 can be explained by increased endogenous concentration of alkylating poisons. Downregulation of ALKBH3 resulted in human non-small-cell lung cancer regression [26], whereas downregulation of ALKBH2 increased the sensitivity of cancer cells to cisplatin, an alkylating-like anticancer drug [27].

The presence of a variety of AlkB homologs in higher organisms raises the question of the biological role of such gene duplication. A similar phenomenon has been observed for another group of DNA repair genes in eukaryotes, namely the recA/RAD51 gene family [28]. The explanation for gene duplication in this case is the role of recA/RAD51 genes in homologous recombination during meiosis. Multiplication of ALKBH genes could be explained by different substrate specificities and/or the physiological state of the cell (phase of cell cycle) leading to gene down- or up-regulation.

We took advantage of the availability of more complete prokaryotic and eukaryotic genome sequences and undertook searches for novel AlkB homologs. Our sequence research involving different sequence queries resulted in 1,943 sequences of different AlkB family members. Most of these sequences have not been previously annotated. Graphical sequence clustering revealed the presence of eight novel AlkB groups. We have chosen *Cyanobacteria* (13 species containing 1–3 AlkB homologs), and *A. thaliana* (13 AlkB homologs found by bioinformatic analysis) as new model organisms for further *in vivo* functional analysis of AlkB homologs. We found that several cyanobacterial AlkBs complemented MMS or CAA treated *E.coli alkB* ^−^ mutant, and increased the survival of M13 (ssDNA) and MS2 (ssRNA) phages after MMS or CAA treatment, indicating efficient repair of 1meA/3meC lesions and etheno adducts in ssDNA and ssRNA.

*In vivo* experiments on the subcellular localization of *A. thaliana* AlkB homologs showed diverse localization of these proteins within the cell, indicating specialized functions in different cell compartments. Relocalization of some homologs upon MMS treatment from cytoplasm to nucleus or from nucleus to nucleoli indicated a role of these proteins in nucleic acids repair.

## Materials and Methods

### Graphical sequence clustering, identification and analysis of the AlkB protein family

Initial PSI-BLAST sequence searches were performed manually using human homologs of *E. coli* AlkB as queries against the NR (non-redundant protein database) at NCBI. These searches allowed us to identify previously unreported plant, fungal and cyanobacterial ABH proteins. Selected sequences were used as queries in further automated PSI-BLAST (5 iterations with 0.01 e-value threshold) and hmmsearch (5 iterations with 0.01 e-value threshold) searches. The cumulative result was manually trimmed to eliminate other 2OG-FeII_Oxy (PF03171) family members. Each group was analyzed separately using secondary structure features as well as the ones based on the presence of conserved residues. All 1943 protein sequences were clustered using CLANS - a graphical java application, based on iterative BLAST all-to-all pairwise searches, that displays the pairwise similarities in either 2D or 3D graphs. As authors claim, “contrary to phylogenetic inference methods this approach uses unaligned sequences and works better the more sequences are provided as an increase in number of pairwise similarities better averages out the chance hits that plague standard BLAST comparisons” [29].

### *In silico* analyses of subcellular localization of *A. thaliana* AlkB homologs

*A. thaliana* AlkB sequences were chosen to predict subcellular protein localization using different available servers (BaCelLo, WoLFPSORT, ProtComp 9.0) and the presence of NLS (cNLS Mapper, NLStradamus) and NES sequences (NetNES 1.1) [30]–[34].

### Bacterial strains, cDNAs, plasmids and media

The strain *Escherichia coli* DH5α (F– Φ80*lac*ZΔM15 Δ(*lac*ZYA-*arg*F) U169 *rec*A1 *end*A1 *hsd*R17 (rK−, mK+) *pho*A *sup*E44 λ– *thi*-1 *gyr*A96 *rel*A1) was used for plasmid propagations. For the purpose of this work *E. coli* AB1157 (*thr-1 ara-14 leuB6* Δ(*gpt-proA)62 lacY1 tsx-33 supE44*_amber_ *galK2 hisG4 rfbD1 mgl-51 rpsL31 kdgK51 xyl-5 mtl-1 argE3 thi-1*) Δ*alkB::kan* (DM12) was constructed according to Datsenco and Wanner [35] and used for complementation and phage survival assays. The pVB1x low copy number plasmid (about 6 copies per cell) was used to prepare constructs harboring cDNA of the investigated *alkB* homologs.

The *Cyanobacteria* strains used in this study are listed in Table 1. The *A. thaliana* cDNA clones were purchased from Arabidopsis Biological Resource Center, DNA Stock Center (Ohio State University) or French Plant Genomic Resource Center (French National Institute for Agricultural Research) (Table 2). In the case of cDNA absence, the genome of *A. thaliana* was isolated with Genome Mini AX Plant (A&ABiot) and then AlkB genes were obtained with the use of appropriate primers in PCR reaction. Transformations were performed according to Sambrook et al. [36]. Liquid media were Luria-Bertani broth (LB) [37] and E medium composed of C salts [38], glucose (0.5%), casamino acids (0.2%), and thiamine (10 µg/ml). The solid media contained 1.5% Difco agar. LCA medium (1% trypton, 0.5% yeast extract, 1% NaCl, 0.25% MgSO_4_×7H_2_O, 2.5 mM CaCl_2_) was solidified with Difco agar at 0.6% [37]. For bacteria bearing antibiotic resistance, carbenicillin (100 µg/ml) and kanamycin (50 µg/ml) were added to the media. Bacteria were grown at 37°C with shaking (250 rpm).

**Table 1:** The *Cyanobacteria* strains with indicated *alkB* homologs (GI numbers according to NCBI database).

Strain	Name used in this study	GI number	Locus ID	Number of *alkB* homologs
*Acaryochloris marina* MBIC11017	aca1	5682958	AM1_4154	2
	aca2	5683725	AM1_4925	2
*Arthrospira maxima* CS-328	art1	209524467	AmaxDRAFT_1993	1
*Cyanothece* sp. PCC7425	cth1	7280326	Cyan7425_5365	3
	cth2	7289383	Cyan7425_3446	3
	cth3	7286719	Cyan7425_0803	3
*Microcoleus chthonoplastes* PCC7420	mic1	7513680	MC7420_2410	2
	mic2	254409401	MC7420_7034	2
*Synechococcus* sp. BL107	sbl1	116065807	BL107_14735	1
*Synechococcus* sp. CC9311	scc1	4260740	sync_1183	1
*Synechococcus* sp. RS9916	srs1	116068221	RS9916_30907	1
*Synechocystis* sp. PCC6803	sis1	2656039	slr7097	3
	sis2	2656221*	slr6021	3
		2656233*	slr6080	3

*indicates various loci with identical sequence.

**Table 2:** *A. thaliana* homologs of *alkB* studied in this work (GI numbers according to NCBI).

Homolog name	Locus name	Gene ID	cDNA
AtALKBH1A	At1g11780	837723	U87108
AtALKBH1B	At3g14140	820631	cDNA not available
AtALKBH1C	At3g14160	820633	C105367
AtALKBH1D	At5g01780	831672	BX830902, BX830679
AtALKBH2	At2g22260	816759	cDNA not available
AtALKBH6	At4g20350	827783	U12618
AtALKBH8A	At1g31600	840048	U18894
AtALKBH8B	At4g02485	827953	U84938
AtALKBH9A	At1g48980	841320	PENTR221-AT1G48980
AtALKBH9B	At2g17970	816307	U61956
AtALKBH9C	At4g36090	829765	S67170
AtALKBH10A	At2g48080	819420	cDNA not available
AtALKBH10B	At4g02940	828132	U17331

### Plasmid construction

The plasmids were constructed by inserting the PCR products encoding the *alkB* gene/cDNA into pVB1x vector. Moreover, the cDNA or the gene of each *A. thaliana alkB* homolog was inserted into pSAT6-EGFP-N1 plasmid (giving fusion of the target protein at the N-terminus of eGFP) and into pSAT6-EGFP-C1 plasmid (fusion at the C-terminus of eGFP) under the tandem CaMV 35S promoter [39].

The *alkB* gene/cDNA was amplified with the primers listed in Tables S1, S2 and S3.

### *A. thaliana* suspension culture, protoplasts isolation and transformation

*A.thaliana* suspension culture ecotype Columbia-0 was grown in the medium consisting of 0.3% Gamborg's B5 Basal Salts Mixture (Sigma), 1× Gamborg's Vitamin Solution (Sigma), 100 µg/L 2.4-D, 0.15% sucrose, pH_KOH_ 5.8, in an environmentally-controlled chamber with constant illumination at 23°C with shaking (120 rpm). *A. thaliana* cell suspension protoplasts were isolated and transformed with 20 µg of plasmid DNA per 10^6^ protoplasts by the polyethylene glycol method as described [40].

### Leptomycin B and methyl methanesulfonate treatment

To recognize whether GFP-fused AlkB proteins are actively shuttled between the nucleus and the cytoplasm, protein localization was investigated in the presence of leptomycin B (LMB, Enzo Life Sciences, 25 ng/ml, 4 h incubation before microscopic observations). LMB inhibits the NES-dependent nuclear export of proteins that is mediated by Exportin 1 (CRM1). To test the leptomycin B activity, the vector expression GFP-NLS-CHS-NES construct was used [41]. The potential changes in subcellular localization of *A. thaliana* AlkB homologs were examined after 30 min of incubation in the presence of 10 mM MMS (Sigma-Aldrich).

### Microscopic observations

Transformed protoplasts were analyzed 17–24 h post transformation by laser scanning confocal microscope (Olympus FV1000 and Nikon C1). Excitation wavelengths and emission filters were 488/510 nm for eGFP, 405/610 nm for chlorophyll autofluorescence and 559/598 nm for staining the mitochondria with 100 nM Mitotracker CMX Ros (Invitrogen).

### Phage survival assay

The phage survival was assayed according to [42]. Two bacteriophages were used, ssDNA phage M13 and ssRNA phage MS2. They were typically propagated in *E. coli* JM105 (F^+^) strain. The *E. coli alkB* ^−^ F^+^ strain bearing pVB1x plasmid expressing the appropriate AlkB homolog (or “empty” pVB1x plasmid as a control) was grown to stationary phase in E-Pro medium supplemented with kanamycin (50 µg/ml) and carbenicillin (100 µg/ml), and diluted 30-fold in fresh medium. 2 mM tolluic acid at OD_600_ = 0.2, and 5 mM MgCl_2_ at OD_600_ = 0.8 were added. 150 µl of bacteria was mixed with 100 µl of phage preparation, incubated for 15 min at 37°C, mixed with 3 ml of warmed to 45°C LCA, and poured onto LB plates. After 10 h of incubation at 37°C the plaques were counted to calculate PFU (Plaque Forming Units per ml).

For phage modification, M13 or MS2 suspensions in C salts were treated with 15 mM MMS or 20 mM chloroacetaldehyde (CAA) for 30 min at 30°C. The reaction was stopped by dilution of the mixtures and PFU was estimated as described above.

### MMS mutagenesis and complementation assay

MMS-induced mutagenesis was assayed as previously [43]. Bacterial cultures grown in E medium to OD_600_ = 0.2 were supplemented with 2 mM toluic acid (Sigma Aldrich). At OD_600_ = 0.5 bacteria were treated with 10 mM MMS for 15 min, centrifuged, washed with C salts supplemented with glucose, casamino acids and thiamine and resuspended in the same volume of fresh medium. To test for mutagenicity, MMS-treated bacteria and non-treated control were diluted 1∶10 in E medium, grown overnight to express mutations, and plated on LB plates for viable cells (one day of incubation at 37°C ) and on E-Arg plates for Arg^+^ revertants (two days of incubation at 37°C ). Following the counts, the frequency of Arg^+^ reversion (number of Arg^+^ revertants per 10^8^ cells) was calculated.

### Statistical analysis

All experiments were carried out at least 4 times, each in duplicate, and the standard deviation error was calculated. Statistically important differences were tested on the basis of Student t-test (p<0.05, two-sided, implication of different variances). Counts were computed and graphs were constructed using calculation sheet of Open Office package.

## Results

### Phylogenetic analysis of cyanobacterial and *A. thaliana* EcAlkB homologs

Latest bioinformatics and functional analysis of AlkB dioxygenases was published in 2009 and concerns only bacterial AlkBs [44]. Here, we performed sequence searches involving different sequence queries that resulted in more than 1943 sequences of different ALKBH family members. Most of these sequences were not previously annotated.

**Figure 1:**
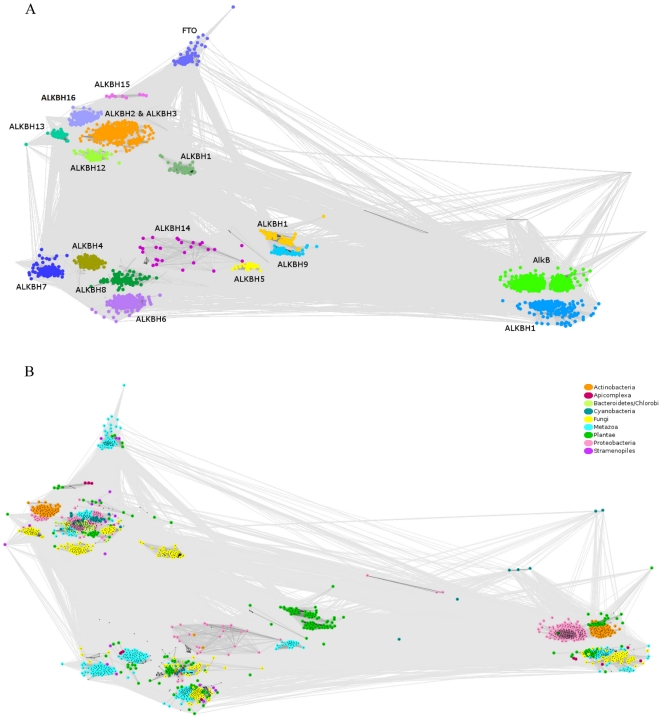
CLANS clustering of 1943 ALKBH proteins. Particular ALKBH groups (A) or taxons (B) are color coded. The groups numbered 9–16 are novel ALKBH family members described in this paper. Color codes are explained in the legend in the upper right corner of (B).

Graphical sequence clustering revealed the presence of novel members of the ALKBH family which we named in concordance with previous publications [15] using the ALKBH acronym together with a number. Our newly identified ALKBH family members are numbered 9 to 16 (Figure 1A). Sequences classified to each group are listed in Fasta files S1, S2, S3, S4, S5, S6, S7, S8, S9, S10, S11, S12, S13, S14, S15, S16, S17, S18, S19, S20.

The clustering image enabled us to observe four major subgroups of AlkB proteins: (i) hALKBH1 and AlkB, (ii) hALKBH2, hALKBH3 and ALKBH11–16, (iii) FTO, and (iv) hALKBH4–8 and ALKBH9–10 (Table 3). Each of these subfamilies includes prokaryotic as well as eukaryotic representatives, except for the FTO clade which encompasses solely eukaryotic sequences. Plant ALKBH proteins are found in subgroupings with metazoan sequences but they also form specific clades (ALKBH9, ALKBH10). Fungi also have specific ALKBH types, ALKBH11, ALKBH12, and ALKBH13, that are almost entirely limited to fungal representatives. Most of the outlying sequences correspond to cyanobacteria. However, no cyanobacterial sequence is found within the bacterial AlkB clade. Each eukaryotic kingdom has a unique ALKBH repertoire, which indicates the importance of ALKBH variety in eukaryotes.

**Table 3:** CLANS clustering of 1943 ALKBH proteins.

Group	Subfamily	Bacteria	Cyanobacteria	Apicomplexa	Stramenopiles	Fungi	Plantae	Metazoa
I	AlkB	X					X	
	ALKBH1	X		X	X	X	X	X
II	ALKBH2 & ALKBH3	X	X	X	X	X	X	X
	ALKBH2_HighGC	X^1^						
III	ALKBH4			X	X		X	X
	ALKBH5							X
	ALKBH6			X	X	X	X	X
	ALKBH7			X	X	X		X
	ALKBH8	X	X	X	X	X	X	X
	ALKBH9						X	
	ALKBH10						X	
II	ALKBH11					X		
	ALKBH12				X^3^	X		
	ALKBH13	X			X	X		
	ALKBH14	X^2^					X^6*^	
	ALKBH15			X			X^6^	
	ALKBH16					X^5^	X^7*^	
IV	FTO				X^4^		X^6^	X^4^

**Figure 2:**
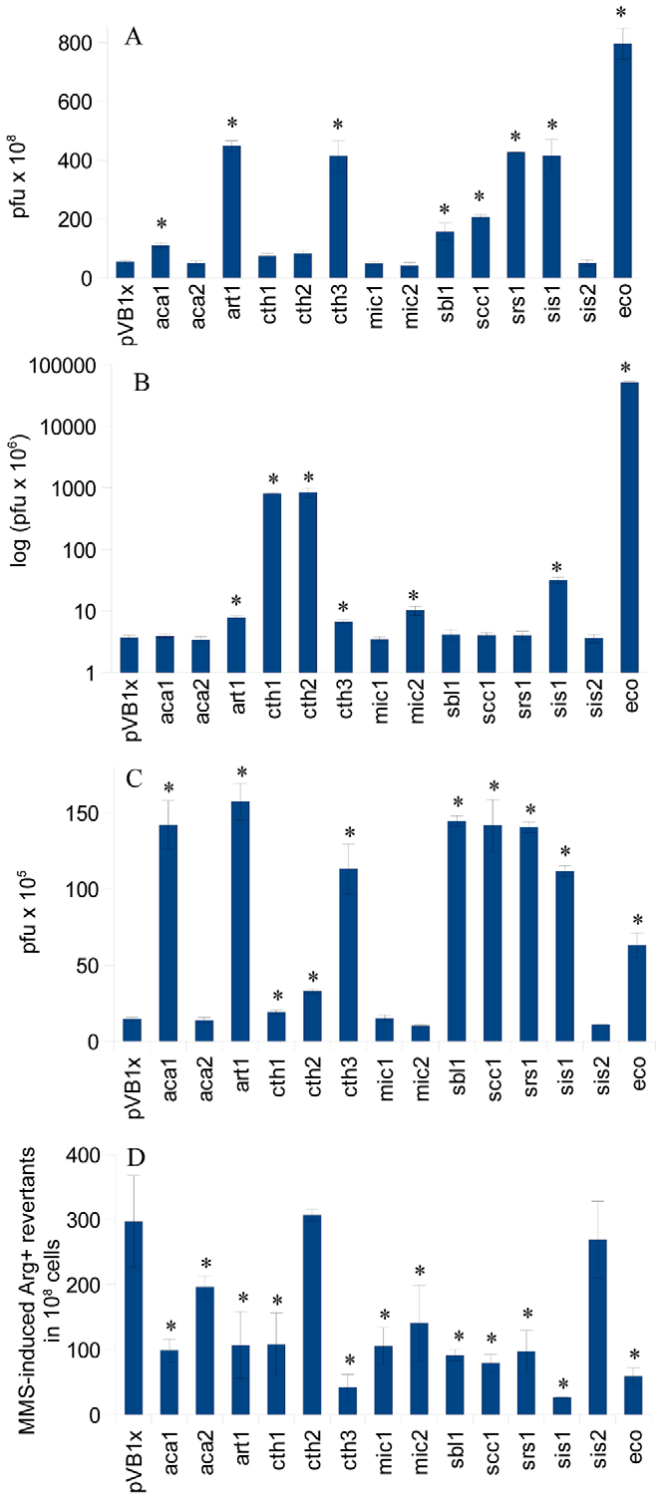
Survival of M13 and MS2 phages and MMS-induced mutagenesis in *E. coli alkB^−^* strain. The survival of MMS (A, C) or CAA (B) treated M13 (A,B) or MS2 (C) phage in the *E. coli alkB* ^−^ strains harboring pVB1x plasmids expressing cyanobacterial AlkB homologs (“empty” vector served as control). Panel D presents the frequency of MMS-induced Arg^+^ revertants in the same strains. Mean values are from at least 4 independent experiments with standard deviation, asterisk indicates statistically significant difference compared to strain with pVB1x plasmid on the basis of Student t-test (p<0.05, two-sided, implication of different variances).

Particular taxons are color coded (according to legend to Figure 2). The groups numbered 9–16 are novel ALKBH family members described in this paper.

1 - Burkholderiales & Actinomycetales; 2 - Rhizobiales and Actinobacteria; 3 – Diatoms; 4 - Diatoms and Brown Algae; 5 – Pezizomycotina; 6 – Green Algae; 7 – Physcomitrella patens; 8 – Chordates (* indicates only one protein).

In the set of sequences that we found there are two types of clans. First is quite compact, with all sequences often coming from one phylum, e.g. ALKBH7, ALKBH9, ALKBH10 or ALKBH11. On the other hand, there is a significant, usually crowded set of clans that have either members belonging to many phyla (ALKBH1, ALKBH6) or that are unusually scattered (ALKBH14).

Table 3 summarizes the distribution of each group of ABHs across kingdoms. Figure 1B shows the taxonomical relationship between the ALKBH groups.

### Survival of M13 and MS2 phages in *E. coli alkB* ^−^ strain harboring plasmids expressing cyanobacterial and *A. thaliana* AlkB homologs

Phage survival assay involving mutagen treated M13 and MS2 phages was performed to test the effect of AlkB homologs on the repair of ssDNA and ssRNA, respectively. Two alkylating agents were tested, the methylating MMS (Figures 2 A, C) and ethylating CAA (Figure 2 B). As could be expected, the *E. coli alkB*, denoted eco, was most effective in increasing the survival of MMS-treated M13 phage. The presence of eco on pVB1x plasmid in *E. coli alkB* ^−^ strain resulted in a 16-fold greater M13 survival (about 8.5×10^10^ PFU/ml) in comparison to the *E. coli alkB* ^−^ bearing the empty pVB1x vector (about 5.0×10^9^ PFU/ml). In the case of art1, cth3, syn3 and sis1 cyanobacterial homologs, M13 survival was 8-fold above the level of empty vector (about 4.5×10^10^ PFU/ml). The syn1 and syn2 increased the phage survival about 3 and 4-fold, respectively. The cth1 and cth2 did not increase the phage survival markedly (no more than 2-fold) but still statistically relevant, as calculated by t-Student test (Figure 2 A).

CAA-treated M13 phage survived almost 14,000-fold better in *E.coli alkB* ^−^ harboring pVB1x eco than in the strain bearing an empty plasmid (about 5.2×10^8^ *vs.* 3.7×10^4^ PFU/ml) (Figure 2 B). Among cyanobacterial AlkB homologs only cth1 and cth2 showed relatively high activity under assay conditions; the strains expressing these homologs increased survival of CAA-treated M13 phage by 221-fold in comparison to the strain bearing the empty vector (about 8.3×10^6^ *vs.* 3.7×10^4^ PFU/ml). However, this result is about 63-fold worse than the one obtained for pVB1x eco plasmid (about 8.3×10^6^ *vs.* 5.2×10^8^ PFU/ml).

MMS-treated MS2 phage survived only 4-fold better in *E. coli alkB* ^−^ harboring pVB1x eco than in the cells bearing an empty plasmid (about 65 *vs.* 17×10^5^ PFU/ml). Interestingly, as many as seven out of 13 cyanobacterial homologs, aca1, art1, cth3, sbl1, scc1, srs1 and sis1, increased MS2 survival by 8-, 9-, 7-, 8-, 8-, 8- and 7- fold, respectively, which is about 2-fold more than in the presence of eco (Figure 2 C). These results show a stronger effect of AlkB homologs on the survival of phage MS2 than of M13.

We did not observe any difference in survival of CAA-treated MS2 phage in *E. coli alkB* ^+^ and *E. coli alkB* ^−^ strains when 20 mM CAA was used.

Summing up the results obtained for two phages and two alkylating agents, cyanobacterial homologs aca1, sbl1 and scc1 increased only the survival of MS2 phage, whereas art1, srs1 and sis1, elevated also though less efficiently, the survival of M13 phage (Figure 2 A and C).

In the case of *A. thaliana* homologs, no AlkB activity of MMS-treated M13 phage was observed toward ssDNA (data not shown).

### MMS-induced mutagenesis in *E. coli alkB* ^−^ mutant supplemented with cyanobacterial AlkB homologs

To test whether cyanobacterial AlkB homologs are able to functionally substitute for the EcAlkB protein, the *E. coli* AB1157Δ*alkB::kan* (DM12) mutant deleted in *alkB* gene was constructed. To monitor the mutagenic potency of MMS-induced 1meA/3meC lesions in *E. coli*, we have chosen *argE3*→Arg^+^ and not *lacZ*→Lac^+^ reversion system since Arg^+^ reversion occurs more efficiently than Lac^+^ reversion (for details see [45]). The pVB1x plasmids harboring different cyanobacterial *alkB* sequences were transformed into AB1157Δ*alkB::kan* strain. Eight out of thirteen cyanobacterial *alkB* homologs expressed from the pVB1x plasmid complemented *alkB* deletion, decreasing the frequency of MMS-induced *argE3*→Arg^+^ reversion to the level observed for *E. coli alkB* ^+^ strain (1.0 Arg^+^ revertants/10^10^ cells) (Figure 2 D). Among the three remaining cyanobacterial *alkB* homologs, cth2 and sis1 did not complement *alkB* mutation at all, whereas aca2 decreased Arg^+^ reversion level by about 40%.

None of the investigated *A. thaliana* homologs complemented the *alkB* deletion in MMS-treated AB1157Δ*alkB* strain (data not shown).

### Subcellular localization of *A. thaliana* AlkB homologs

The first approach focused on the *in silico* analysis of *A. thaliana* AlkB protein localization. According to this analysis, the majority of AlkB homologs localized in the nucleus and/or cytoplasm. However, the *in silico* prediction of *A. thaliana* AlkB homologs subcellular localization varied depending on the program used, and in most of the cases was not experimentally confirmed (Table S4). The exception was the AtALKBH1D homolog, which was predicted to localize in chloroplasts and to consist of potential nuclear localization signal (NLS). Localization in chloroplasts and in the nucleus was confirmed *in vivo*, indicating that NLS found *in silico* may be a functional nuclear localization signal. The sequences of *A. thaliana* AlkB homologs were also searched for potential nuclear localization signals (NLS). Every protein with predicted NLS localized *in vivo* at least partially in the nucleus, but there were homologs accumulating in the nucleus, such as AtALKBH8A and AtALKH6, with no predicted NLS (Table S5).

**Figure 3:**
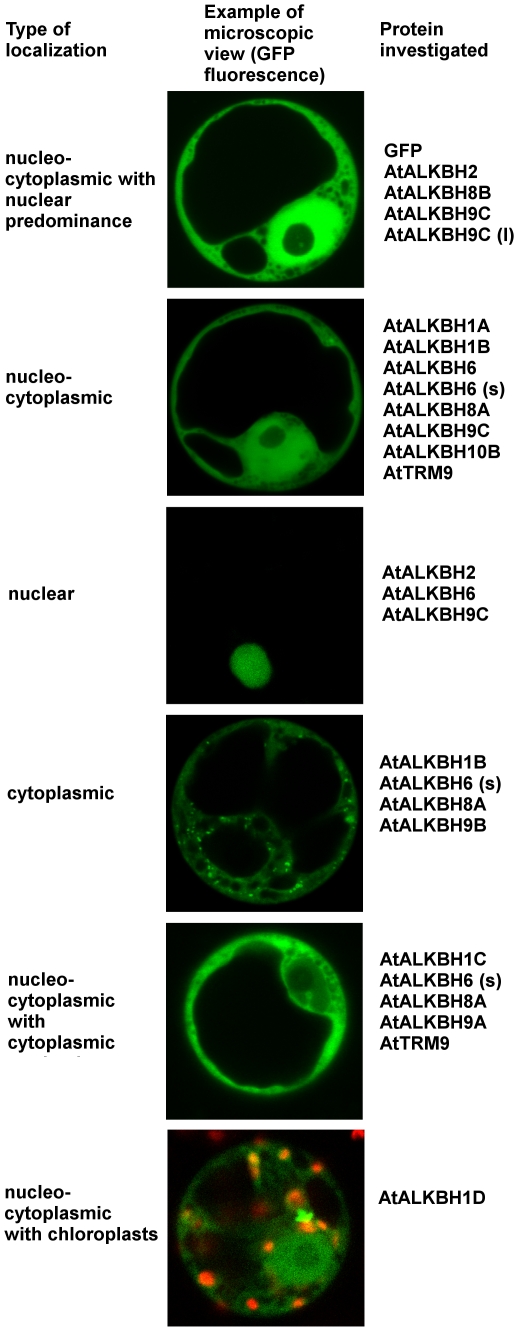
Subcellular localization of GFP-tagged AlkB homologs. *A. thaliana* protoplasts from cell suspension culture were transfected with constructs expressing the indicated proteins in N- and C- terminal fusion with GFP and visualized by confocal laser-scanning microscopy. AlkB homologs represent 6 types of subcellular localization. The image for AtALKBH1D homolog localization is merged with the red autofluorescence of chlorophyll (orange color comes from overlay of GFP and chlorophyll fluorescence). For comparison GFP fluorescence alone (pSAT6-eGFP) was also analyzed. N - nucleus, Nu - nucleous, Ch - chloroplasts, V - vacuole. For more details see Supplementary Data.

*In vivo* experiments showed that *A. thaliana* AlkB homologs present six different localization types. They can be localized equally or not equally in the cytoplasm and nucleus, exclusively in the nucleus or cytoplasm and partially in the chloroplasts (Figure 3; Figure S1). It should be mentioned that only five of the investigated proteins were localized independently on GFP fusion type. Furthermore, some of the homologs showed two types of localization in different ratio (Table S6).

### Relocalization of *A. thaliana* AlkB homologs after MMS treatment or LMB inhibition

**Figure 4:**
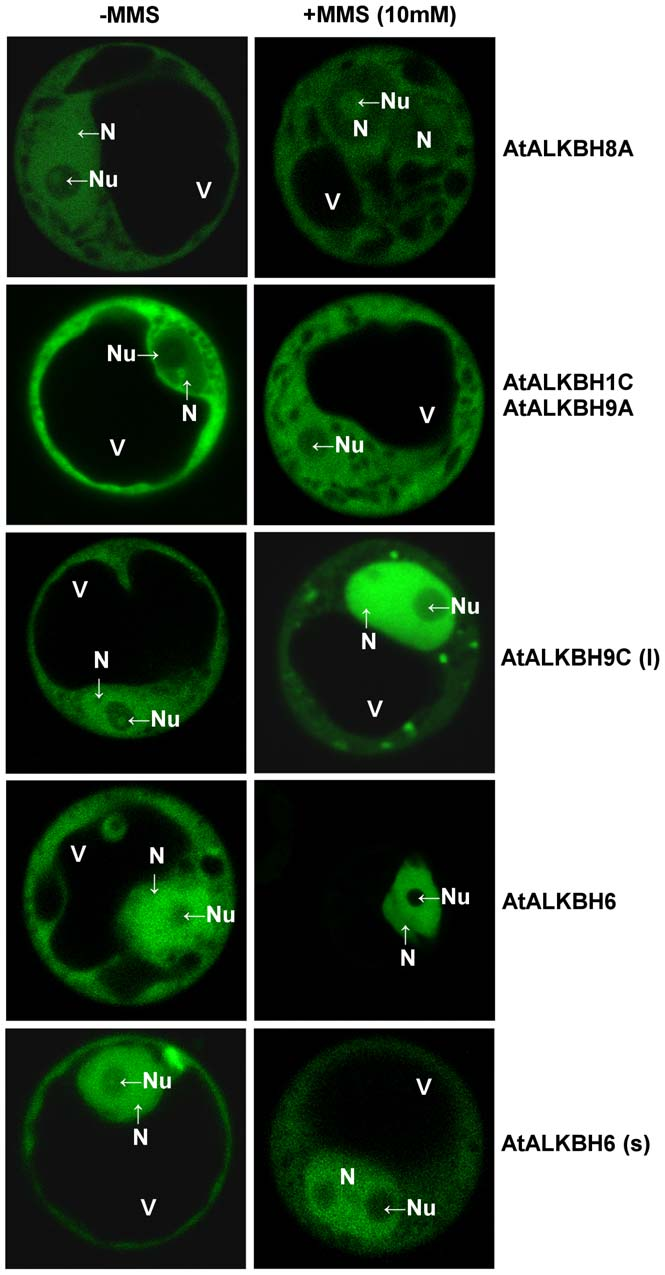
Relocalization of *A. thaliana* AlkB homologs after MMS treatment. *A. thaliana* suspension culture protoplast transiently transfected with GFP-fused AlkB homologs were incubated with 10 mM MMS for 30 min and analyzed using confocal laser-scanning microscopy. While the localization of AtALKBH1A, AtALKBH1B, AtALKBH8B, AtALKBH9C homologs and AtTRM9 protein did not change during the MMS-treatment, the localization of AtALKBH1C, AtALKBH6 (s), AtALKBH9A and AtALKBH9C (l) shifted to more nuclear; AtALKBH6 localized exclusively in the nucleus. In the case of the AtALKBH8A, both cytoplasmatic and nuclear localizations were observed. The scan demonstrate the main localization of presented homologs.

The influence of alkylating agent, MMS, on the subcellular localization of *A. thaliana* AlkB homologs was also examined. In most of the cases we observed relocalization of GFP-tagged proteins to the nucleus. The exception was the AtALKBH8A homolog that after MMS treatment localized in both, the cytoplasm and nucleolus (Figure 4). We have not confirmed the influence of MMS on the localization of AtALKBH1A, AtALKBH1B, AtALKBH8B, and AtALKBH9C homologs and of AtTRM9.

**Figure 5:**
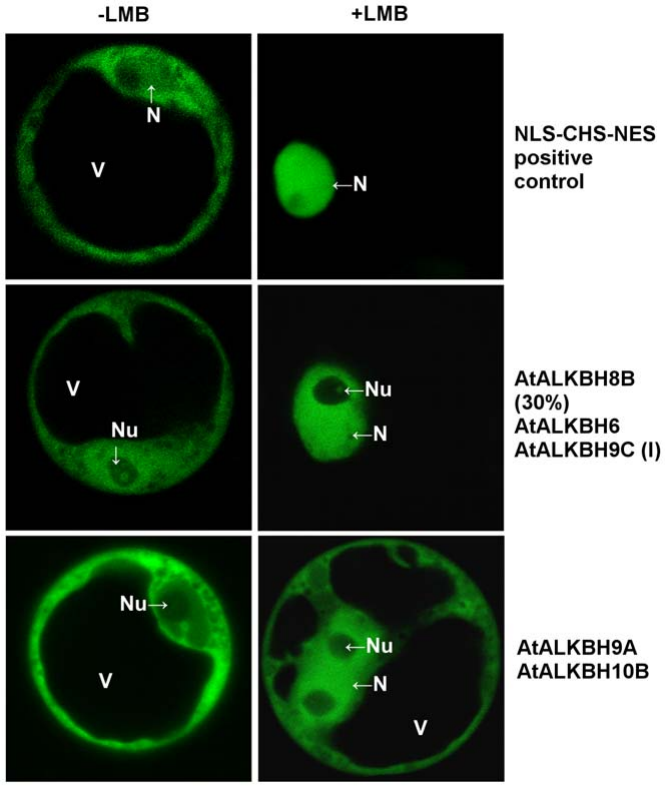
Relocalization of *A. thaliana* nucleo-cytoplasmic AlkB homologs after LMB inhibition of nuclear export. Protoplasts transfected with GFP-AlkB homologs were incubated with LMB for 4 h and analyzed for GFP fluorescence using confocal laser-scanning microscopy. As a positive control plasmid expressing GFP-NLS-CHS-NES was used. All of the protoplasts transfected with AtALKBH6 and AtALKBH9C(l), and 30% of those transfected with AtALKBH8B changed their localization to exclusively nuclear after LMB treatment. In the case of AtALKBH9A and AtALKBH10B the signal in the nucleus was more intense after incubation with LMB, and LMB did not inhibit AtALKBH1A, AtALKBH8A, AtALKBH9C, AtALKBH6s and AtTRM9 export. The scans demonstrate the main localization of presented homologs.

In the case of nucleo-cytoplasmic location of AlkB proteins, the question arose whether export of AlkB proteins from the nucleus to the cytoplasm is CRM1 dependent. First of all, we analyzed the sequences of *A. thaliana* AlkB homologs *in silico* in order to find the predicted NES sequences (Table S5). Then, the homologs at least partially localizing in the nucleus were selected and their localization upon LMB treatment was checked (Figure 5, Figure S2). Although the AtALKBH1A homolog showed the predicted NES sequence, its localization did not change after LMB treatment. Moreover, LMB did not inhibit AtALKBH8A, AtALKBH9C, AtALKBH6s and AtTRM9 export. Inversely, AtALKBH6 and AtALKBH9Cl changed their localization to exclusively nuclear after LMB treatment even though they have no predicted NES. In the case of AtALKBH9A and AtALKBH10B, the NES sequences were found and the signal in the nucleus was more intense after incubation with LMB. Furthermore, upon LMB inhibition, 30% of protoplasts transfected with AtALKBH8B showed only nuclear localization (Figure S2). These results demonstrate that all the mentioned homologs are nucleo-cytoplasmic shuttling proteins and that their shuttling is at least partly controlled by the CRM1 receptor.

## Discussion

In this study we have performed a bioinformatics analysis of protein sequence database allowing identification of new prokaryotic and eukaryotic ALKBH dioxygenases, homologs of *Escherichia coli* AlkB dioxygenase involved in the repair of alkylation lesions in DNA and RNA. AlkB proteins are present in almost all organisms and, what is more, there are multiple dioxygenases present, especially in higher eukaryotes; hence, the question arises about the functions of the variety of these ALKBH proteins. The regulation of ALKBHs has previously been studied in tumors, where at least three of them are upregulated and participate in cancer development. HeLa cells are not a good model for ALKBH research since, like in tumors, most of the proteins analyzed are highly expressed in this cell line. Also knockout mice in *Alkbh*s are not especially valuable for investigations of the biological role of ALKBHs. Out of genes analysed only *Alkbh1* knockout was affected in basic developmental processes, suggesting a pivotal role for ALKBH1 in the dealkylation process [46], [47].

In order to categorize the available sequences of different AlkB family members we performed a sophisticated bioinformatic clustering which revealed the presence of 1,943 sequences of different AlkB family members and eight novel clans of AlkB homologs (Figure 1). These findings allowed to subdivide AlkB proteins into four major groups including known and identified by us ALKBH proteins and clearly show that there is a unique set of AlkBs for each kingdom of life, due to the different physiological requirements (Table 3).

Results obtained *in silico* prompted us to examine alternative models for investigation of ALKBH functions. We decided to apply *in vivo* biochemical analysis in *Cyanobacteria*, a simple model of an organism possessing 1–3 AlkB homologs. These gene products were able to complement MMS-treated *E. coli alkB* ^−^ mutant and increase the survival of phages M13 and MS2 after MMS or CAA treatment, indicating the efficient repair of 1meA/3meC lesions and etheno adducts, respectively, in DNA and RNA. Moreover, cyanobacterial AlkBs appear to be very active against lesions in ssRNA, while EcAlkB favors ssDNA repair (compare Figure 2 A and C). In concert, AlkB proteins from plant RNA viruses have been shown to remove methyl lesions from nucleic acids with higher activity towards RNA than DNA substrates [48]. Discovered within the present study high repair activity of cyanobacterial AlkB towards RNA seems to support previously reported *in vitro* experiments, where EcAlkB and hALKBH3 restored the activity of methylated mRNA and tRNA [49]. None of the 11 *A. thaliana* homologs complemented *E. coli alkB* null mutant (data not shown). Presumably, plant AlkBs are simply inactive when expressed in bacteria, which is in agreement with our preliminary *in vitro* biochemical tests.

Our *in vivo* experiments on the subcellular location of 13 *A. thaliana* AlkB homologs showed diverse localization of these proteins, indicating specialized functions in different cell compartments. Nuclear localization of At2g22260 and its high similarity to hALKBH2 indicate possible role of this protein in dsDNA repair. The localization of At5g01780 (AtALKBH1D) partially in chloroplasts indicates probable involvement of this protein in organellar system of alkylation lesion repair, which resembles the role of hALKBH1 in mitochondria [17]. Additionally, for some AlkB homologs we observed translocation from the cytoplasm to the nucleus or from the nucleus to the nucleoli after MMS treatment, confirming their role in the repair of alkylation lesions in nuclear or nucleoli components, including nucleic acids. The relocation of *A. thaliana* AlkB homolog At1g31600 (AtALKBH8) to the nucleolus after MMS treatment may corroborate its role in RNA metabolism in this nuclear compartment. Moreover, our observation that nucleo-cytoplasmic AtTRM9 protein did not change its subcellular localization after MMS treatment confirms the expectations of Leihne et al. [22] that this protein, in contrast to its yeast counterpart, is not a component of the DNA damage response machinery. Location of some homologs almost exclusively in the cytoplasm suggests their role in the repair of other than DNA alkylated substrates.

A very recent study of Korvald et al. [50] shows absolutely surprising interaction of *Schizosaccharomyces pombe* AlkB homolog Ofd2 with histones. Moreover, its biological activity is stimulated by histones suggesting the role of histones as a prime substrate. In our opinion when looking for the biological role of the ALKBHs, we should abandon analyzing these enzymes as involved mainly in DNA repair. We find the hypothesis that the multiplicity of the ALKBHs is due to their involvement in the metabolism of large nucleo-protein complexes, such as histones [50] or, as recently suggested, ribosomes [51], to be very probable. Dealkylation of the substrates catalyzed by this class of dioxygenases is an one-step repair with no high energy costs. Thus, it is much more effective to repair a single alkyl lesion in histone/ribosome than to degrade these huge complexes and exchange all the components. In the light of this hypothesis, we postulate that high ALKBH levels in different types of human cancer serve not only in DNA repair, since this function can be fulfilled by other DNA repair systems, but are also involved in the repair of mentioned above large nucleo-protein complexes.

## Supporting Information

Figure S1 ***In vivo*** **localization of AlkB** ***A. thaliana*** **homologs.** Subcellular localization of GFP-tagged AlkB homologs. *A. thaliana* protoplasts from cell suspension culture were transfected with constructs expressing the indicated proteins in N- and C- terminal fusion with GFP and visualized by confocal laser-scanning microscopy. The image for AtALKBH1D homolog localization is merged with the red autofluorescence of chlorophyll (orange color comes from overlay of GFP and chlorophyll fluorescence). N - nucleus.
(PDF)

Figure S2 **Relocation of** ***A. thaliana*** **AlkB homologs upon LMB inhibition.** Relocalization of *A. thaliana* nucleo-cytoplasmic AlkB homologs after LMB inhibition of nuclear export. Protoplasts transfected with constructs expressing AlkB-GFP fusions were incubated with LMB for 4 h and analyzed for GFP fluorescence using confocal laser-scanning microscopy. All of the protoplasts transfected with AtALKBH6 and AtALKBH9C(l), and 30% of those transfected with AtALKBH8B changed their localization to exclusively nuclear after LMB treatment. In the case of AtALKBH9A and AtALKBH10B the signal in the nucleus was more intense after incubation with LMB, and LMB did not inhibit AtALKBH1A, AtALKBH8A, AtALKBH9C, AtALKBH6s and AtTRM9 export. N - nucleus.
(PDF)

Fasta File S1 **Particular AlkB protein sequence homologs.**
(FASTA)

Fasta File S2 **Particular ALKBH1 protein sequence homologs.**
(FASTA)

Fasta File S3 **Particular ALKBH2&3 protein sequence homologs.**
(FASTA)

Fasta File S4 **Particular ALKBH2 protein sequence homologs.**
(FASTA)

Fasta File S5 **Particular ALKBH2 protein sequence homologs with high GC content.**
(FASTA)

Fasta File S6 **Particular ALKBH3 protein sequence homologs.**
(FASTA)

Fasta File S7 **Particular ALKBH4 protein sequence homologs.**
(FASTA)

Fasta File S8 **Particular ALKBH5 protein sequence homologs.**
(FASTA)

Fasta File S9 **Particular ALKBH6 protein sequence homologs.**
(FASTA)

Fasta File S10 **Particular ALKBH7 protein sequence homologs.**
(FASTA)

Fasta File S11 **Particular ALKBH8 protein sequence homologs.**
(FASTA)

Fasta File S12 **Particular ALKBH9 protein sequence homologs.**
(FASTA)

Fasta File S13 **Particular ALKBH10 protein sequence homologs.**
(FASTA)

Fasta File S14 **Particular ALKBH11 protein sequence homologs.**
(FASTA)

Fasta File S15 **Particular ALKBH12 protein sequence homologs.**
(FASTA)

Fasta File S16 **Particular ALKBH13 protein sequence homologs.**
(FASTA)

Fasta File S17 **Particular ALKBH14 protein sequence homologs.**
(FASTA)

Fasta File S18 **Particular ALKBH15 protein sequence homologs.**
(FASTA)

Fasta File S19 **Particular ALKBH16 protein sequence homologs.**
(FASTA)

Fasta File S20 **Particular FTO protein sequence homologs.**
(FASTA)

Table S1 **Primers used to set PCR reaction for introduction of cyanobacterial** ***alkB*** **homologs into pVB1x vector.**
(DOC)

Table S2 **Primers used to set PCR reaction for introduction of** ***A. thaliana alkB*** **homologs into pVB1x vector.**
(DOC)

Table S3 **Primers used to set PCR reaction for introduction** ***A. thaliana alkB*** **homologs into pSAT6-GFP vector.**
(DOC)

Table S4 ***In silico*** **localization prediction of** ***A. thaliana*** **AlkB homologs.** The scores for particular predictions are indicated in brackets. For Softberry protComp the maximal score accounts for 10. In the case of Wolfpsort the numbers indicate the closest homologs of particular subcellular localization (N – nucleus, C –cytoplasm, CYT – cytoskeleton, CH – chloroplasts, MCH – chloroplast membrane, MT – mitochondria, PL – plastids, GA – Golgi apparatus, ER – endoplasmatic reticulum, P – plasmalemma, V – vacuole, SEC – secreted protein)
(DOC)

Table S5 **Prediction of NLS and NES sequences in** ***A. thaliana*** **AlkB homologs.** NLS sequences found in one of the programs used were checked in the second program with decreased cut-off. When found, they are written in parenthesis. Amino acids found in both programs are underlined. (NF – not found)
(DOC)

Table S6 ***In vivo*** **localization of** ***A. thaliana*** **AlkB homologs.** The level of GFP fluorescence was arbitrally marked as: strong (++), medium (+), weak (+−). It was also detected in nucleolus vacuole (*) and as aggregates (A). Particular homologs showed ambiguous localization which is indicated as a percentage of protoplasts with indicated GFP signals.
(DOC)
