# Supplementary material for: Novel AlkB Dioxygenases—Alternative Models for In Silico and In Vivo Studies
Source: PLoS One. 2012 Jan 24;7(1):e30588. doi: 10.1371/journal.pone.0030588 (PMC3265494; doi:10.1371/journal.pone.0030588)
Supplement: Figure S1 — In vivo localization of AlkB A. thaliana homologs. Subcellular localization of GFP-tagged AlkB homologs. A. thaliana protoplasts from cell suspension culture were transfected with constructs expressing the indicated proteins in N- and C- terminal fusion with GFP and visualized by confocal laser-scanning microscopy. The image for AtALKBH1D homolog localization is merged with the red autofluorescence of chlorophyll (orange color comes from overlay of GFP and chlorophyll fluorescence). N - nucleus. (PDF) [file pone.0030588.s001.pdf]

<table>
<tr><th rowspan="3">AlkB homolog</th><th colspan="6">The type of GFP fusion</th></tr>
<tr><th colspan="3">ORF-GFP</th><th colspan="3">GFP-ORF</th></tr>
<tr><th>GFP</th><th>DIA</th><th>merged</th><th>GFP</th><th>DIA</th><th>merged</th></tr>
<tr><td>AtALKBH1A</td><td colspan="3">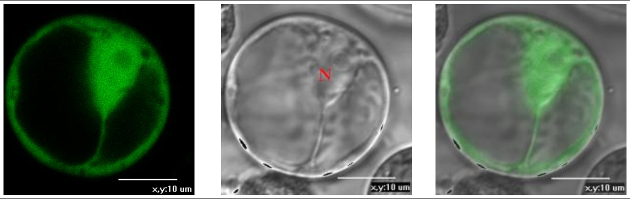</td><td colspan="3">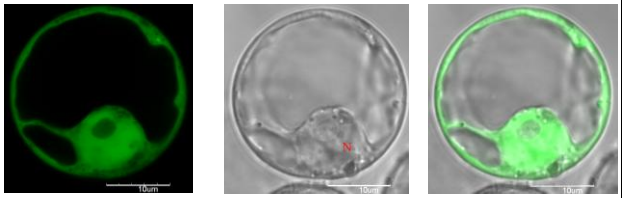</td></tr>
<tr><td>AtALKBH1B</td><td colspan="3">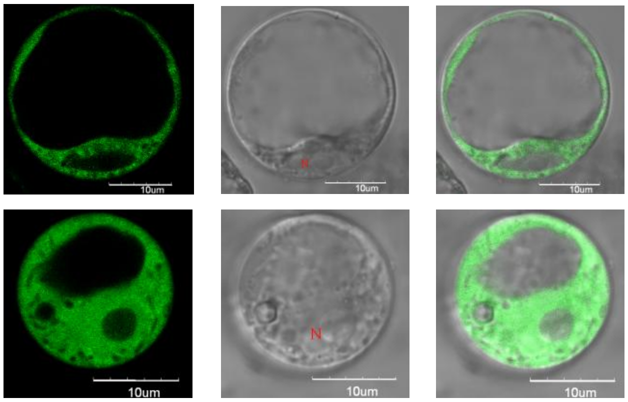</td><td colspan="3">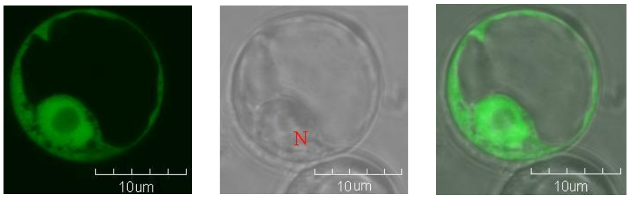</td></tr>
<tr><td>AtALKBH1C</td><td colspan="3">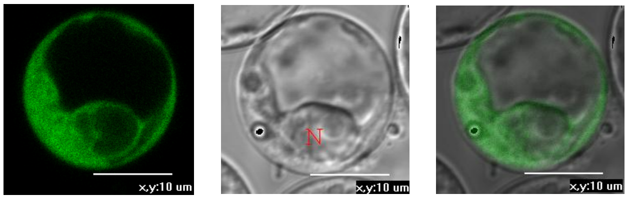</td><td colspan="3">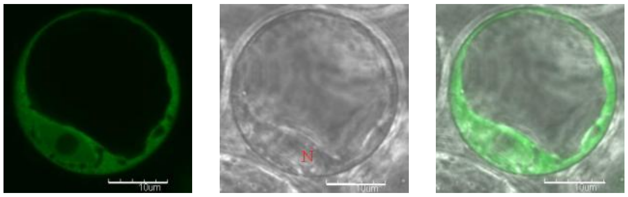</td></tr>
<tr><td>AtALKBH1D</td><td colspan="3">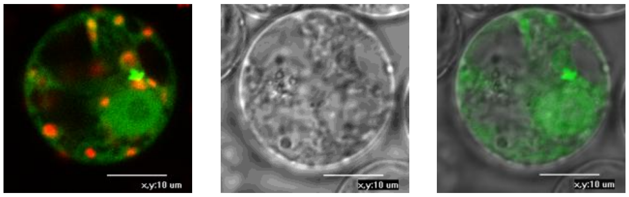</td><td colspan="3">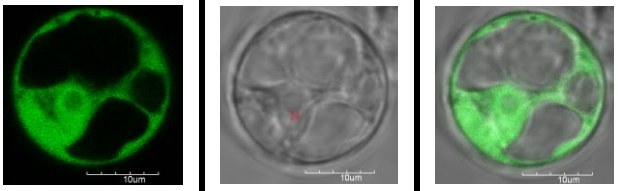</td></tr>
<tr><td>AtALKBH2</td><td colspan="3">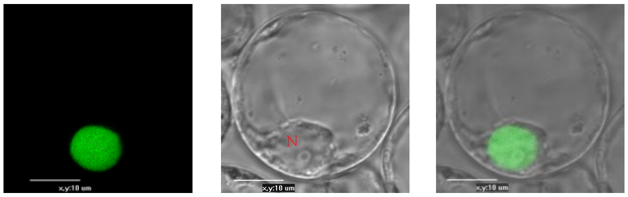</td><td colspan="3">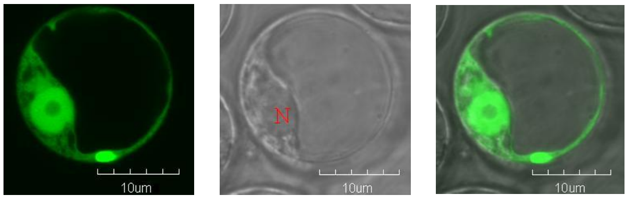</td></tr>
</table>

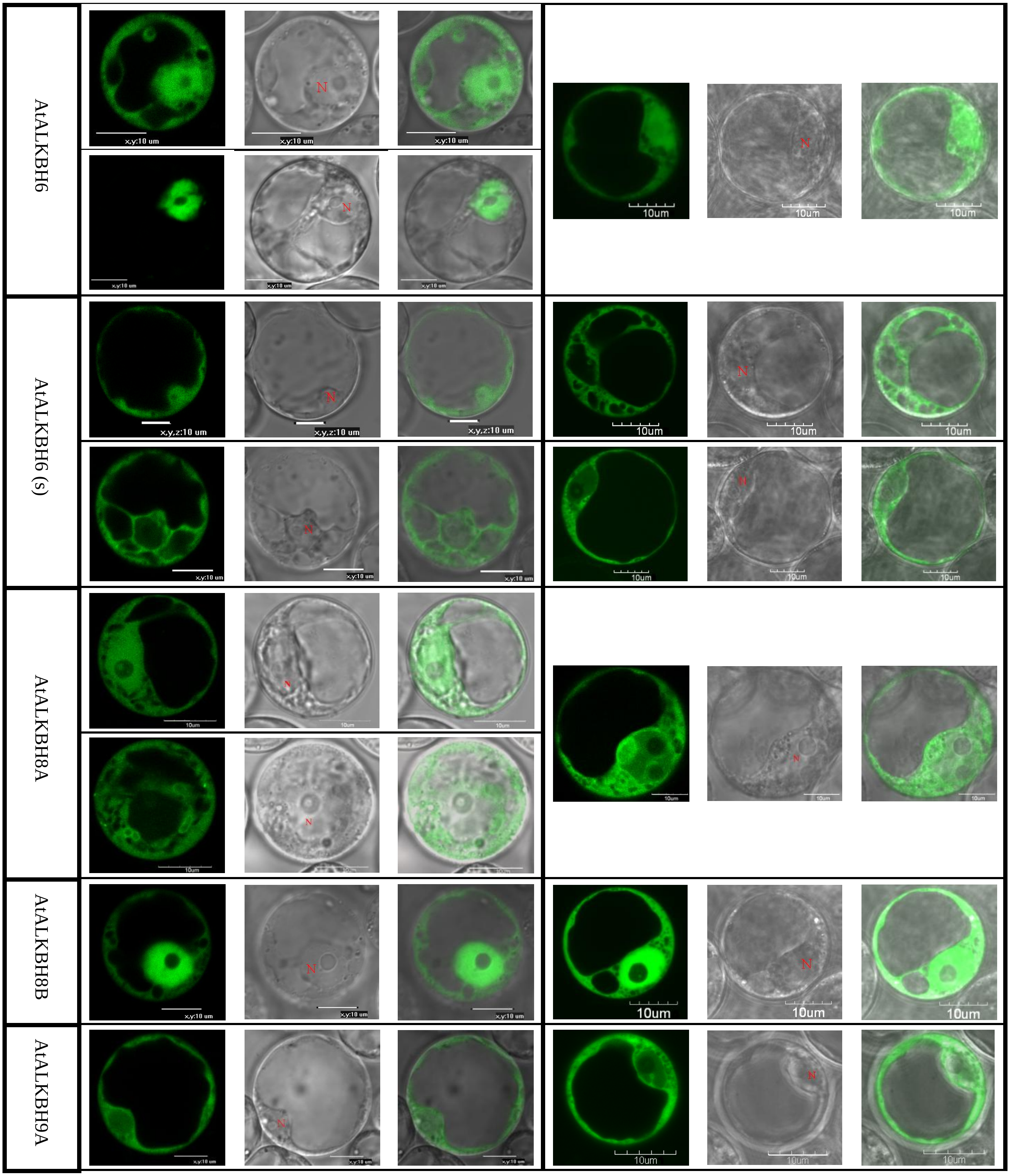

AtALKBH6
AtALKBH6 (s)
AtALKBH8A
AtALKBH8B
AtALKBH9A
N
x,y:10 um
x,y,z:10 um
10um

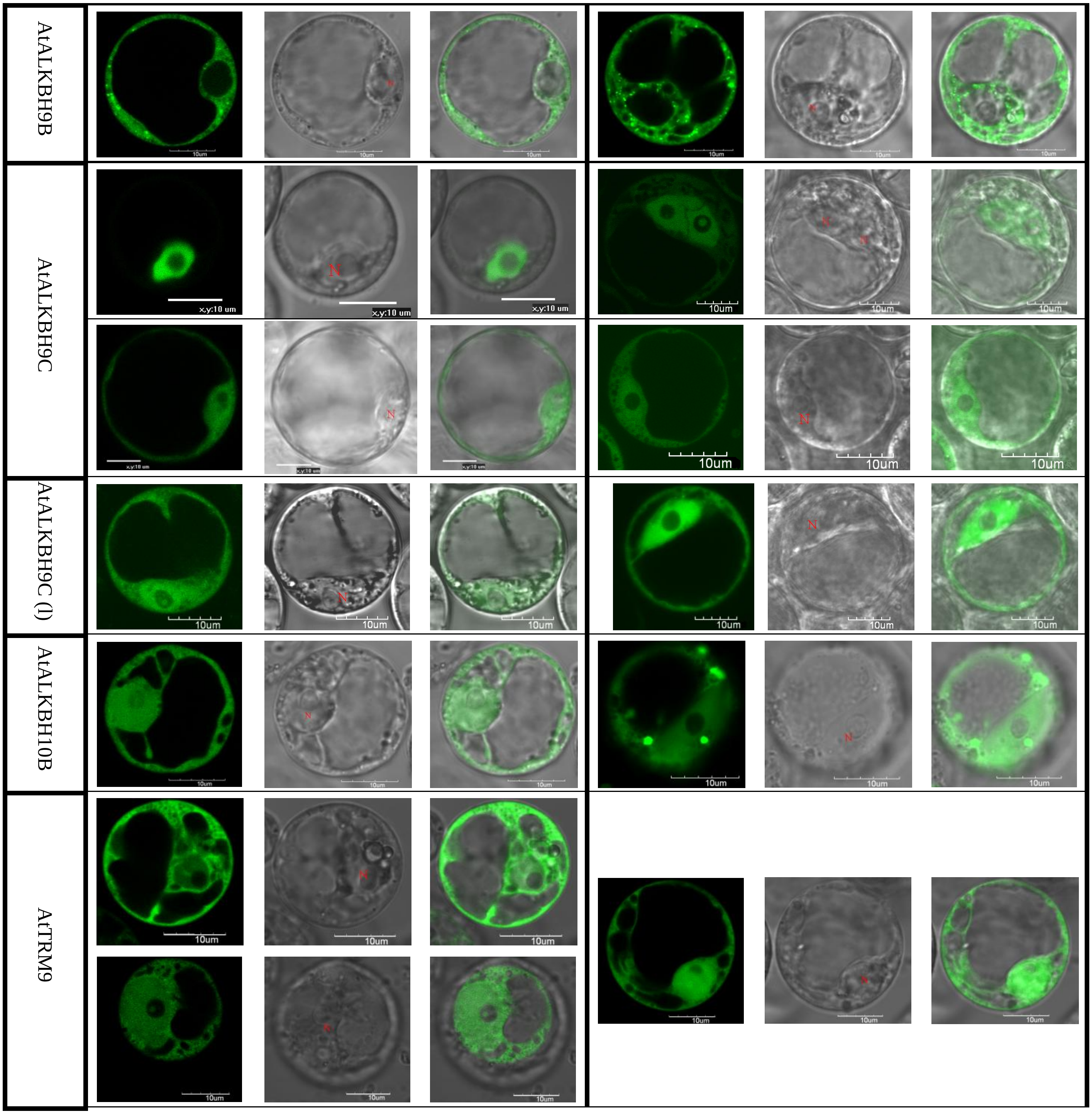
AtALKBH9B
AtALKBH9C
AtALKBH9C (J)
AtALKBH10B
AtTRM9
N
10um
x,y:10 um
