# Supplementary figures and images for: Novel AlkB Dioxygenases—Alternative Models for In Silico and In Vivo Studies
Source: PLoS One. 2012 Jan 24;7(1):e30588. doi: 10.1371/journal.pone.0030588 (PMC3265494; doi:10.1371/journal.pone.0030588)

| AlkB homolog  |     | GFP                                                                                 |  | DIA                                                                                  |  | merged                                                                                |  |
|---------------|-----|-------------------------------------------------------------------------------------|--|--------------------------------------------------------------------------------------|--|---------------------------------------------------------------------------------------|--|
| AtALKBH6      |     | 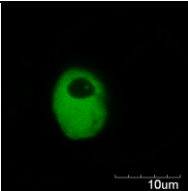   |  | 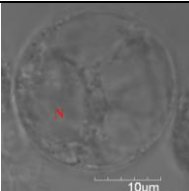   |  | 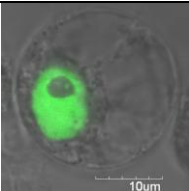   |  |
| AtALKBH8B     | 70% | 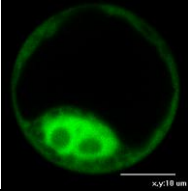   |  | 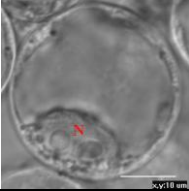   |  | 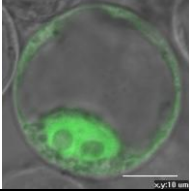   |  |
|               | 30% | 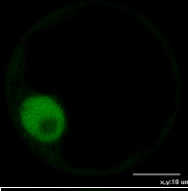   |  | 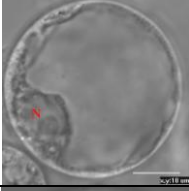   |  | 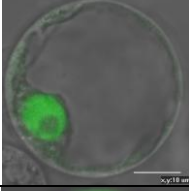   |  |
| AtALKBH9A     |     | 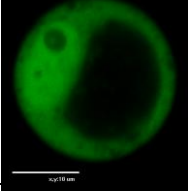  |  | 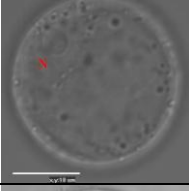  |  | 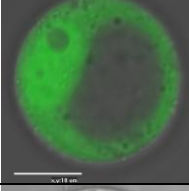  |  |
| AtALKBH9C (l) |     | 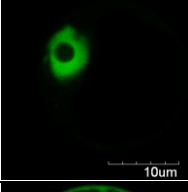 |  | 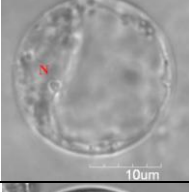 |  | 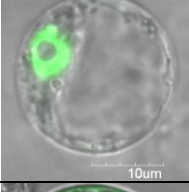 |  |
| AtALKBH10B    |     | 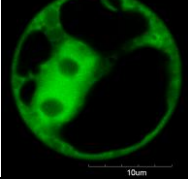 |  | 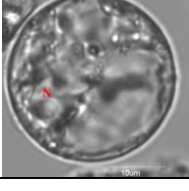 |  | 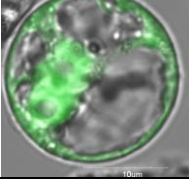 |  |

Supplement: Figure S2 — Relocation of A. thaliana AlkB homologs upon LMB inhibition. Relocalization of A. thaliana nucleo-cytoplasmic AlkB homologs after LMB inhibition of nuclear export. Protoplasts transfected with constructs expressing AlkB-GFP fusions were incubated with LMB for 4 h and analyzed for GFP fluorescence using confocal laser-scanning microscopy. All of the protoplasts transfected with AtALKBH6 and AtALKBH9C(l), and 30% of those transfected with AtALKBH8B changed their localization to exclusively nuclear after LMB treatment. In the case of AtALKBH9A and AtALKBH10B the signal in the nucleus was more intense after incubation with LMB, and LMB did not inhibit AtALKBH1A, AtALKBH8A, AtALKBH9C, AtALKBH6s and AtTRM9 export. N - nucleus. (PDF) [file pone.0030588.s002.pdf]
