# Supplementary material for: Novel AlkB Dioxygenases—Alternative Models for In Silico and In Vivo Studies
Source: PLoS One. 2012 Jan 24;7(1):e30588. doi: 10.1371/journal.pone.0030588 (PMC3265494; doi:10.1371/journal.pone.0030588)
Supplement: Table S1 — Primers used to set PCR reaction for introduction of cyanobacterial alkB homologs into pVB1x vector. (DOC) [file pone.0030588.s023.doc]

| Cyanobacterial *alkB* homolog | Primer (forward and reverse) | Primer sequence (forward and reverse) | Restriction endonuclease |
| --- | --- | --- | --- |
| aca1 | aca1Nde | CAGCTCATATGCCTGATGCAGAGGTGTTG | NdeI |
|  | aca1Xho | ATCGACTCGAGTTACTGAATGACTCGAAAAGT | XhoI |
| aca2 | aca2Nde | CAGCTCATATGAGTTTTGCCCCTCCAGAT | NdeI |
|  | aca2BamH | ATCGAGGATCCTTAGCTGATGATACTTCTAAA | BamHI |
| art1 | art1Nde | CAGCTCATATGAGACTGTGGGATAATCAG | NdeI |
|  | art1Xho | ATCGACTCGAGCTATGATTTTACCATTCTAAA | XhoI |
| cth1 | cth1Nde | CAGCTCATATGACCGCCTATACCCAGCTC | NdeI |
|  | cth1Xho | ATCGACTCGAGTTAAACCATAACCTTACGAAA | XhoI |
| cth2 | cth2Nde | CAGCTCATATGTTCAGTCATCAGCTAAGT | NdeI |
|  | cth2Xho | ATCGACTCGAGTCAATCCCCCAAAATAACCTT | XhoI |
| cth3 | cth3Nde | CAGCTCATATGGCTGAACAGCTTCACCTC | NdeI |
|  | cth3Xho | ATCGACTCGAGTTACGCATGGACTATCCGGAAG | XhoI |
| mic1 | mic1Nde | CAGCTCATATGTATTACAATTTTTTCCGT | NdeI |
|  | mic1Xho | ATCGACTCGAGTCAAATGATTTTTCTAAAAGT | XhoI |
| mic2 | mic2Nde | CAGCTCATATGATACCCTCGTCAAAATCG | NdeI |
|  | mic2Xho | ATCGACTCGAGTTATTTATACGGAAACACAAC | XhoI |
| syn1 | syn1Nde | CAGCTCATATGGACCCAGTCATCAGCGCT | NdeI |
|  | syn1BamH | ATCGAGGATCCTTACCCATGAAAGAAATGGCG | BamHI |
| syn2 | syn2Nde | CAGCTCATATGAGCTGGAACCATCGTGTT | NdeI |
|  | syn2Xho | ATCGACTCGAGTCACGGTGCCAATTTGGGCTG | XhoI |
| syn3 | syn3Nde | CAGCTCATATGACGGGCCTGCCTTGGACG | NdeI |
|  | syn3Xho | ATCGACTCGAGTCAGCGTCCCCCTTTGGTGGC | XhoI |
| sis1 | sis1Nde | CAGCTCATATGTTGATTAAACAACTCAGCCTTTTTGA | NdeI |
|  | sis1Xho | ATCGACTCGAGTTATTGAATAAAGCGGAAGGTTAAAT | XhoI |
| sis2 | sis2Nde | CAGCTCATATGTTTAAAAAATTAGGTGAA | NdeI |
|  | sis2Xho | ATCGACTCGAGTTATAAAACTTTTCTAATACT | XhoI |
| eco | EcalkBN | CAGCTCATATGTTGGATCTGTTTGCCGA | NdeI |
|  | EcalkBX | ATCGACTCGAGTTATTCTTTTTTACCTGCCTG | XhoI |
