# Supplementary material for: Novel AlkB Dioxygenases—Alternative Models for In Silico and In Vivo Studies
Source: PLoS One. 2012 Jan 24;7(1):e30588. doi: 10.1371/journal.pone.0030588 (PMC3265494; doi:10.1371/journal.pone.0030588)
Supplement: Table S2 — Primers used to set PCR reaction for introduction of A. thaliana alkB homologs into pVB1x vector. (DOC) [file pone.0030588.s024.doc]

| *A.thaliana alkB* homolog | Primer (forward and reverse) | Primer sequence (forward and reverse) | Restriction endonuclease |
| --- | --- | --- | --- |
| At**ALK**BH1A | At1g11up | CTGAATCATATGTACGAATCGGCGAA | NdeI |
|  | At1g11dn | CAATGCCTCGAGTCAGAAAACTTGCCTGAT | XhoI |
| AtALKBH1C | At3g14up | CTGAATCATATGTATTGTGATCTTGTG | NdeI |
|  | At3g14dn | CAATGCGGATCCCTAATACTGCCTAAAAGT | BamHI |
| AtALKBH1D.1 | At5g1up | CGTAGCCATATGAGGCCTGGAATGGTG | NdeI |
|  | At5g1dn | CAATGCCTCGAGTTAGAAATGCCTAAGAGT | XhoI |
| AtALKBH1D.2 | At5g2up | CGTAGCCATATGATGTGCCTTGGGCGA | NdeI |
|  | At5g2dn | CAATGCCTCGAGTTAGAAATGCCTAAAAGT | XhoI |
| AtALKBH6 | At4g20up | CTGAATCATATGAAAAGGGTCTTGTTC | NdeI |
|  | At4g20dn | CAATGCGGATCCCTAGAATCTGAAGAGATT | BamHI |
| AtALKBH8A | At1g31up | CTGAATCATATGGGCTGGCCATGGGCT | NdeI |
|  | At1g31dn | CAATGCCTCGAGTTACATTTGTTGCTGAGA | XhoI |
| AtALKBH8B | At4g02up | CTGAATCATATGGACGAAGAAGCAGAG | NdeI |
|  | At4g02dn | CAATGCCTCGAGTTAGGCTTGACATAGCTT | XhoI |
| AtALKBH9A | At1g48up | CTGAATGAATTCGATGGACAACTATTCTTTTATC | EcoRI |
|  | At1g48dn | CAATGCGGATCCCTAAAATACATGGTCAGA | BamHI |
| AtALKBH9B | At2g17up | CTGAATCATATGGAAAACGATCCATTTCTCCGG | NdeI |
|  | At2g17dn | CAATGCCTCGAGTTAACCGTAGTTTCTTCTACTAGG | XhoI |
| AtALKBH9C | At4g36up | CTGAATCATATGGAACCAAATTATGAG | NdeI |
|  | At4g36dn | CAATGCGGATCCTTACCCGACAGGAAGCGG | BamHI |
| AtALKBH10B | At4g029u | CTGAATCATATGACGATTGCGGCAGCGCCAGCA | NdeI |
|  | At4g029d | CAATGCCTCGAGTTAACCTACAGTGATCACAGGCTC | XhoI |
| AtTRM9 | At1g36up | CTGAATCATATGATTTTGGATGTTTTAAGAACTTTTTCTA | NdeI |
|  | At1g36dn | CAATGCCTCGAGTTAATCTTGGTTTAAAGCTTCTTTCTG | XhoI |
