# Supplementary material for: Novel AlkB Dioxygenases—Alternative Models for In Silico and In Vivo Studies
Source: PLoS One. 2012 Jan 24;7(1):e30588. doi: 10.1371/journal.pone.0030588 (PMC3265494; doi:10.1371/journal.pone.0030588)
Supplement: Table S3 — Primers used to set PCR reaction for introduction A. thaliana alkB homologs into pSAT6-GFP vector. (DOC) [file pone.0030588.s025.doc]

| *A.thaliana alkB* homolog | | Primer (forward and reverse) | Primer sequence (forward and reverse) | Restriction endonuclease |
| --- | --- | --- | --- | --- |
| AtALKBH1A | | 1g11Sup | ACACGAGCTCAATGTACGAATCGGCGAAC | SacI |
|  | | 1g11Supb | ACACGAGCTCAAATGTACGAATCGGCGAAC | SacI |
|  | | 1g11Sdn | ACACGGATCCCGAAAACTTGCCTGAT | BamHI |
| AtALKBH1B | | 3g14Sup | ACACGAGCTCAATGGCAACGACCAATTAC | SacI |
|  | | 3g14Supb | ACACGAGCTCAAATGGCAACGACCAATTAC | SacI |
|  | | 3g14Sdn | ACACGGATCCCGACGACCTGGTTGAA | BamHI |
| AtALKBH1C | | 3g1416Su | ACACGAGCTCAATGTATTGTGATCTTGTG | SacI |
|  | | 3g1416Sb | ACACGAGCTCAAATGTATTGTGATCTTGTG | SacI |
|  | | 3g1416Sd | ACACGGATCCCATACTGCCTAAAAGT | BamHI |
| AtALKBH1D | | 5g01Sup0 | ACACCTCGAGCATGTTGAACTCTATCCAT | XhoI |
|  | | 501Sup0b | ACACCTCGAGCTATGTTGAACTCTATCCAT | XhoI |
|  | | 5g01Sdn0 | ACACGTCGACTGAAATGCCTAAAAGT | SalI |
| AtALKBH2 | | 2g22Sup | ACACGAGCTCAATGACGAATCCACTTAAT | SacI |
|  | | 2g22Supb | ACACGAGCTCAAATGACGAATCCACTTAAT | SacI |
|  | | 2g22Sdn | ACACGGATCCCCAAAACAAGCCTGAA | BamHI |
| AtALKBH6 | | 4g20Sup | ACACGAGCTCAATGAAAAGGGTCTTGTTC | SacI |
|  | | 4g20Supb | ACACGAGCTCAAATGAAAAGGGTCTTGTTC | SacI |
|  | | 4g20Sdn | ACACGGATCCCGAATCTGAAGAGATT | BamHI |
| AtALKBH6 (s) | 4g20Supk | | ACACGAGCTCAATGCCGCATCAAGATGGA | SacI |
|  | 4g20Sukb | | ACACGAGCTCAAATGCCGCATCAAGATGGA | SacI |
|  | 4g20Sdn | | ACACGGATCCCGAATCTGAAGAGATT | BamHI |
| AtALKBH8A | | 1g31Sup | ACACGAGCTCAATGGGCTGGCCATGGGCT | SacI |
|  | | 1g31Supb | ACACGAGCTCAAATGGGCTGGCCATGGGCT | SacI |
|  | | 1g31Sdn | ACACGGATCCCCATTTGTTGCTGAGA | BamHI |
| AtALKBH8B | | 4g02Sup | ACACGAGCTCAATGGACGAAGAAGCAGAG | SacI |
|  | | 4g02Supb | ACACGAGCTCAAATGGACGAAGAAGCAGAG | SacI |
|  | | 4g02Sdn | ACACGGATCCCGGCTTGACATAGCTT | BamHI |
| AtALKBH9A | | 1g48Sup | ACACAAGCTTCATGGACAACTATTCTTTTATC | HindIII |
|  | | 1g48Supb | ACACAAGCTTCGATGGACAACTATTCTTTTATC | HindIII |
|  | | 1g48Sdn | ACACGGATCCCAAATACATGGTCAGA | BamHI |
| AtALKBH9B | | 2g17up | ACACCTCGAGCATGGAAAACGATCCATTTCTCCGG | XhoI |
|  | | 2g17upb | ACACCTCGAGCTATGGAAAACGATCCATTTCTCCGG | XhoI |
|  | | 2g17dnS | ACACCCGCGGTAACCGTAGTTTCTTCTACTAGGACG | SacII |
| AtALKBH9C | | 4g36Sup | ACACGAGCTCAATGGAACCAAATTATGAG | SacI |
|  | | 4g36Supb | ACACGAGCTCAAATGGAACCAAATTATGAG | SacI |
|  | | 4g36Sdn | ACACGGATCCCCCCGACAGGAAGCGG | BamHI |
| AtALKBH9C (l) | | 4g36Sup | ACACGAGCTCAATGGAACCAAATTATGAG | SacI |
|  | | 4g36Supb | ACACGAGCTCAAATGGAACCAAATTATGAG | SacI |
|  | | 4g36Sdnd | ACACGGATCCCGACGTTGTCGGAGCT | BamHI |
| AtALKBH10A | | 2g48up | ACACCTCGAGCATGGCTGAAACGCCGGCTTCTCCT | XhoI |
|  | | 2g48upb | ACACCTCGAGCTATGGCTGAAACGCCGGCTTCTCCT | XhoI |
|  | | 2g48dnS | ACACCCGCGGTAACTCACTCCTATCTCTGGCGACGA | SacII |
| AtALKBH10B | | 4g029u | ACACCTCGAGCATGACGATTGCGGCAGCGCCAGCA | XhoI |
|  | | 4g29ub | ACACCTCGAGCTATGACGATTGCGGCAGCGCCAGCA | XhoI |
|  | | 4g029dS | ACACCCGCGGTAACCTACAGTGATCACAGGCTCAGA | SacII |
| AtTMR9 | | 1g36up | ACACGAGCTCAATGATTTTGGATGTTTTAAGAACTTTTTCTA | SacI |
|  | | 1g36upb | ACACGAGCTCAAATGATTTTGGATGTTTTAAGAACTTTTTCTA | SacI |
|  | | 1g36dn | ACACGGATCCCATCTTGGTTTAAAGCTTCTTTCTG | BamHI |
