# Supplementary material for: Novel AlkB Dioxygenases—Alternative Models for In Silico and In Vivo Studies
Source: PLoS One. 2012 Jan 24;7(1):e30588. doi: 10.1371/journal.pone.0030588 (PMC3265494; doi:10.1371/journal.pone.0030588)
Supplement: Table S4 — In silico localization prediction of A. thaliana AlkB homologs. The scores for particular predictions are indicated in brackets. For Softberry protComp the maximal score accounts for 10. In the case of Wolfpsort the numbers indicate the closest homologs of particular subcellular localization (N – nucleus, C –cytoplasm, CYT – cytoskeleton, CH – chloroplasts, MCH – chloroplast membrane, MT – mitochondria, PL – plastids, GA – Golgi apparatus, ER – endoplasmatic reticulum, P – plasmalemma, V – vacuole, SEC – secreted protein) (DOC) [file pone.0030588.s026.doc]

| **Homolog AlkB** | **BaCelLo** | **Wolfpsort** | **protComp** |
| --- | --- | --- | --- |
|  | www.biocomp.unibo.it/bacello/ | *wolfpsort.org* | www.softberry.com |
| AtALKBH1A | N | C (5.0); N (4.0); CYT (3.0); CH (2.0) | C (8.7) |
| AtALKBH1B | N | CH (6.0); C (3.0); SEC (3.0); V (1.0) | CH (5.1) |
| AtALKBH1C | N | N (7.0); CH (3.0); CYT (3,0) | N (6,8) |
| AtALKBH1D | CH | CH (10.5); CH I MT (7.5); MT (3.5) | CH (4.2) |
| AtALKBH2 | N | N (8.5); C and N (5.0); CH (2.0); PL (1.5); GA I PL (1,5) | N (5.0) |
| AtALKBH6 | N | CH (5.0); C (2.0); SEC (2.0); V (2.0); MT (1.0); PL (1,0) | SEC (6.8) |
| AtALKBH6 (s) | N | CH (7.0); MT (3.0); C (2.0); ER (2.0) | P (3,9) |
| AtALKBH8A | N | N (7.0); CH (2.0); C (2.0); CYT (2.0) | MCH (3.4) |
| AtALKBH8B | N | C (8.0); N (2.0); PL (2.0); CH (1.0) | CH (3,8) |
| AtALKBH9A | N | N (5.0); C (4.0); CYT (3.0); CH (2.0) | MCH (4.2) |
| AtALKBH9B | N | N (14.0) | N (7.3) |
| AtALKBH9C | N | N (11.0); C (1.0); SEK (1,0) | N (8.2) |
| AtALKBH9C (l) | N | N (13.0) | N (6.3) |
| AtALKBH10A | N | N (12.0); CH (1.0) | N (6.4) |
| AtALKBH10B | N | C (6.0); CH (5.0); N (3.0) | ER (3,7) |
| AtTRM9 | N | CH (7.0); MT (5.0); N (1,0) | MCH (6.0) |
