# Supplementary material for: Novel AlkB Dioxygenases—Alternative Models for In Silico and In Vivo Studies
Source: PLoS One. 2012 Jan 24;7(1):e30588. doi: 10.1371/journal.pone.0030588 (PMC3265494; doi:10.1371/journal.pone.0030588)
Supplement: Table S5 — Prediction of NLS and NES sequences in A. thaliana AlkB homologs. NLS sequences found in one of the programs used were checked in the second program with decreased cut-off. When found, they are written in parenthesis. Amino acids found in both programs are underlined. (NF – not found) (DOC) [file pone.0030588.s027.doc]

| **AlkB homologue** | Prediction of NLS sequence | | Prediction of NES sequence |
| --- | --- | --- | --- |
|  | **cNLS Mapper**  Cut-off 7.0 | **NLStradamus**  Cut-off 0.6 | **NES**  (NetNES 1.1) |
| AtALKBH1A | 20- RRAEKKYKLYYEQDSKFSRKKKLPKPI | NF (35 – RKKKL, with cut-off 0.5 ) | 43- IDLSELLDFNLI |
| AtALKBH1B | NF | 226 - KKK | NF |
| AtALKBH1C | NF | NF | 262- LTLESGDVLLF |
| AtALKBH1D | NF | 51 – RRRRR | NF |
| AtALKBH2 | 259- GPAKKRLKRS | 257 - PAKKRLKR | NF |
| AtALKBH6 | NF | NF | NF |
| AtALKBH6 (s) | NF | NF | NF |
| AtALKBH8A | NF | NF | NF |
| AtALKBH8B | NF | NF | 101- LESVDL |
| AtALKBH9A | 82- INVKRKRDFV | NF (69 – SRKQRTHIRAINVKRKR, with cut-off 0.3 ) | 101- LEGLEL |
| AtALKBH9B | NF | 169 – RRGELKKRT | 431- IEPLPLDL |
| AtALKBH9C | 180- KTPEKRKLSREERERYRFMNVKKMKVF | NF (180 – EKRKLSREER, with cut-off 0.3) | NF |
| AtALKBH9C (l) | NF | NF | NF |
| AtALKBH10A | 382- PAPKRLDAGTGVFLPWTPPVSRKPAKHLPP | NF (403 – RKPAKHL, with cut-off 0.3) | 674- YLHMLGV |
| AtALKBH10B | NF | 536 - LPPRAQKK | 257- LLD |
| AtTRM9 | NF | NF | NF |
