# Supplementary material for: Novel AlkB Dioxygenases—Alternative Models for In Silico and In Vivo Studies
Source: PLoS One. 2012 Jan 24;7(1):e30588. doi: 10.1371/journal.pone.0030588 (PMC3265494; doi:10.1371/journal.pone.0030588)
Supplement: Table S6 — In vivo localization of A. thaliana AlkB homologs. The level of GFP fluorescence was arbitrally marked as: strong (++), medium (+), weak (+−). It was also detected in nucleolus vacuole (*) and as aggregates (A). Particular homologs showed ambiguous localization which is indicated as a percentage of protoplasts with indicated GFP signals. (DOC) [file pone.0030588.s028.doc]

| **AlkB homolog** | **ORF-GFP** | | | **%** | **GFP-ORF** | | | **%** |
| --- | --- | --- | --- | --- | --- | --- | --- | --- |
|  | nucleus | nucleous | cytoplasm |  | nucleus | nucleous | cytoplasm |  |
| AtALKBH1A | ++ | +- | + |  | ++ | +- | + |  |
| AtALKBH1B | - | - | + | 65 | + | - | + |  |
|  | + | - | + | 35 |  |  |  |  |
| AtALKBH1C | +- | - | ++ |  | + | - | ++ |  |
| AtALKBH1D | + | - | + | A | ++ | +- | + |  |
| AtALKBH2 | + | + | - |  | ++ | +- | + |  |
| AtALKBH6 | + | - | + | 65 | + | + | + |  |
|  | + | - | - | 35 |  |  |  |  |
| AtALKBH6 (s) | + | - | + | 85 | - | - | + | 75 |
|  | - | - | + | 15 | +- | - | + | 25 |
| AtALKBH8A | + | - | + | 80 |  |  |  | A |
|  | - | - | + | 20 | + | - | ++ |  |
| AtALKBH8B | ++ | - | + |  | ++ | - | + |  |
| AtALKBH9A | + | - | ++ |  | + | - | ++ |  |
| AtALKBH9B | - | - | + | A | - | - | + | A |
| AtALKBH9C | + | - | - | 90 | ++ |  | + | 70 |
|  | + |  | + | 10 | + | -* | + | 30 |
| AtALKBH9C (l) | + | - | + |  | ++ | - | + |  |
| AtALKBH10A | ambiguous localization | | | | | | | |
| AtALKBH10B | + | + | + |  | + | - | + | A |
| AtTRM9 | + |  | ++ | 70 | + | - | + |  |
|  | + | -* | + | 30 |  |  |  |  |
| pSAT6-eGFP | + | - | + |  | + | - | + |  |
